# Exogenously Applied Cytokinin Altered the Bacterial Release and Subsequent Stages of Nodule Development in Pea *Ipd3/Cyclops* Mutant

**DOI:** 10.3390/plants12030657

**Published:** 2023-02-02

**Authors:** Elizaveta S. Kantsurova (Rudaya), Alexandra N. Ivanova, Polina Y. Kozyulina, Elena A. Dolgikh

**Affiliations:** 1 All-Russia Research Institute for Agricultural Microbiology, Podbelsky Chausse 3, Pushkin, 196608 St. Petersburg, Russia; 2 Komarov Botanical Institute RAS, Prof. Popov St., 2, 197376 St. Petersburg, Russia; 3 Research Park, St. Petersburg State University, Universitetskaya Emb. 7-9, 199034 St. Petersburg, Russia

**Keywords:** *Pisum sativum*, transcriptomic data, nodule, differentiation, bacteria release, IPD3/CYCLOPS transcription factor, cytokinin, mutant, gene expression

## Abstract

Regulation of plant hormonal status is one of the major targets of symbiotic signaling during nodule formation in legume plants. However, the genetic and hormonal networks that regulate transition to differentiation of nodules are not well-characterized in legume plants. Analysis of plant mutants forming nodules impaired in rhizobial infection allowed us to identify some regulators involved in the control of the later stages of nodule development. In the current work, we extend our earlier studies on the influence of exogenously applied cytokinin on the later stages of nodule morphogenesis using pea *sym33* (*ipd3/cyclops*) mutants impaired in the gene encoding IPD3/CYCLOPS transcription factor. One of the noticeable effects of the influence of exogenously applied cytokinin on nodules in the *sym33-3* mutant was an increasing size of these structures. Cytokinin treatment was shown to stimulate bacterial release and increase the percentage of infected cells in nodules. To explore the role of possible regulators of nodule differentiation, we performed searching in pea transcriptome. The transcriptome study in pea *P. sativum* revealed the importance of the CCS52 regulator, EFD transcription factor, SYMREM regulator, RSD, the MADS-domain/AGL, and SHORT INTERNODE/STYLISH gene families encoding transcription factors in the control of nodule differentiation. Analysis of the expression patterns was verified by real-time PCR in response to exogenously applied cytokinin treatment.

## 1. Introduction

Plant hormones play key roles in the interactions between legume plants and nitrogen-fixing bacteria rhizobia [1,2,3]. The perception of bacterial lipochitooligosaccharide signals, the Nod factors, initiates a signaling cascade that induces rhizobial infection in the epidermis and activation of cortical cell division that leads to the nodule formation. During the infection process and nodule initiation, regulation of plant hormonal status is one of the major targets of symbiotic signaling. Numerous studies have shown that the activation of cytokinin signaling in response to Nod factor perception [4,5,6,7,8,9,10,11] followed by a local increase in auxin in the cortex due to the inhibition of polar auxin transport (PAT) and auxin biosynthesis stimulation may be crucial for the control of nodule primordium formation [12,13,14,15,16,17,18,19,20,21]. Legumes belonging to the inverted repeat-lacking clade (IRLC) such as *Medicago truncatula*, *Pisum sativum,* and *Vicia sativa* form indeterminate nodules with persistent apical meristem. According to the fine map of nodule morphogenesis in *M. truncatula*, induced hormonal changes seem to be related to the stages from I to IV, when the divisions are induced in pericycle and endodermis, followed by the divisions in C5 and C4 cell layers and C3 layer (future meristem) [22]. Starting from the stage V and up to the stage VI, a multi-layered meristem is formed and starts to produce daughter cells for the nodule tissues. This allows one to distinguish the appearing structure as a new organ, the nodule, and further developmental steps may be related to nodule differentiation. Using an *Arabidopsis* Cyclin B1 reporter *AtCyclB1.1::GUS*, which is active during mitosis, the appearance of a meristem was distinguished from endoreduplicating cells of C5 and C4 layers in nodules [22]. However, the genetic and hormonal networks that regulate the transition to differentiation of nodules are not well-characterized in legume plants.

During nodule development, the size of the meristem is tightly regulated, since the appearance of new sets of meristematic cells is balanced with the exit of the number of cells from the mitotic cell cycle. The layers that are subsequently formed from daughter cells of meristem become infected with bacteria. The growth and differentiation of infected plant cells result in their enlargement, which is mediated by endoreduplication cycles (replication of the genome without mitosis and cytokinesis) [23]. The mitotic inhibitor CELL-CYCLE SWITCH 52 (CCS52) is involved in the regulation of endoreduplication, which is an essential step during normal differentiation of functional, nitrogen-fixing nodules [24]. Recent studies established that components of the plant DNA topoisomerase VI such as SUNER1 and VAG1 are also involved in the control of endoreduplication and required for cell growth of rhizobia-infected cells [25,26]. Moreover, the differentiation of bacteroides in indeterminate nodules demonstrates remarkable similarity to host cell differentiation [23]. This suggests that bacteroid differentiation and endoreduplication are mediated by plant factors in nodules.

Analysis of plant mutants forming nodules impaired in infection by rhizobia allowed the identification of some regulators involved in the control of later stages of nodule development. As an example, analysis of *M. truncatula* mutants defective in the *MtNF-YA1* (*MtHAP2-1*) gene showed that bacterial release and microRNA169 may be important for transition of the daughter cells to endoreduplication and subsequent bacterial infection [27,28]. It is proposed that microRNA169 may restrict the *MtNF-YA1 (MtHAP2-1)* expression to the nodule meristematic zone that is essential for the differentiation of cells in the infection zone of the nodule [27]. This points towards the possible link between bacterial release and the production of microRNA169 in plant cells.

Another transcriptional regulator, the IPD3 (called CYCLOPS in *Lotus japonicus*), participates in signaling cascade and is essential for bacteria release from infection threads in legumes such as *Pisum sativum, L. japonicus,* and *M. truncatula* [29,30,31,32,33,34]. In nodules of *ipd3/cyclops* mutants, the central tissues remain non-infected, which results in defects in subsequent plant cells and bacteroid differentiation. Therefore, these mutants may be considered as a useful model for the investigation of later stages of nodule formation related to the differentiation of plant cell and bacterial cell.

Mutational analysis has recently shown that abnormalities in rhizobial infection and subsequent plant cell and bacteroid differentiation in pea *ipd3/cyclops* mutants (*sym33-2, sym33-3*) coincide with altered pattern of cytokinin response and immunolocalization [35]. Moreover, in *L. japonicus cyclops* mutant defective in bacterial release and subsequent steps of nodule development, a high level of auxin still exists in the central part of forming nodules in contrast to wild-type nodules, where it is restricted to the peripheral tissues [13]. However, the physiological pathways through which cytokinin and auxin might influence later stages of nodule development are poorly characterized and require further study.

In the current work, we extend our earlier studies on the influence of exogenously applied cytokinin on the later stages of nodule morphogenesis using pea *ipd3/cyclops* (*sym33*) mutants impaired in the IPD3/CYCLOPS transcription factor. Here, we focused on the ultrastructural changes in mutant nodules under the influence of exogenous cytokinin, as well as performing a more detailed analysis of genes related to bacterial release, accommodation, and plant differentiation.

## 2. Results and Discussion

### 2.1. The Influence of Cytokinin on Morphology and Ultrastructure of Nodules in Pea Sym33-3 Mutant

The pea *sym33-3* mutant impaired in the gene orthologous to *IPD3* in *Medicago truncatula* and *CYCLOPS* in *L. japonicus* forms a dramatically decreased amount of small ineffective nodules compared to wild-type plants [30,32,34]. These rare nodules remain uninfected due to the blocking of bacterial release from the infection threads. Therefore, the IPD3/CYCLOPS transcriptional factor is involved in the control of intracellular bacterial accommodation and seems to induce a set of downstream regulators important for subsequent stages of nodule morphogenesis, many of which are still not known.

Indeed, our previous analysis indicated that the pattern of cytokinin distribution in *sym33-3* and *sym33-2* mutant nodules may reflect changes in regulation of the later stages of symbiosis development in pea [35]. Using the RNAseq analysis of pea *sym33-3* mutant treated with 10 µM exogenously applied cytokinin, we have previously found about 540 genes that were similarly up-regulated in nodules of mutant treated with cytokinin as well as in wild-type nodules compared to mutant nodules using Log2 fold change value > 2 and *p* adjusted value < 0.05 [36]. It demonstrates that this approach may be useful to find new regulators stimulated by cytokinin at later stages of nodulation.

**Figure 1:**
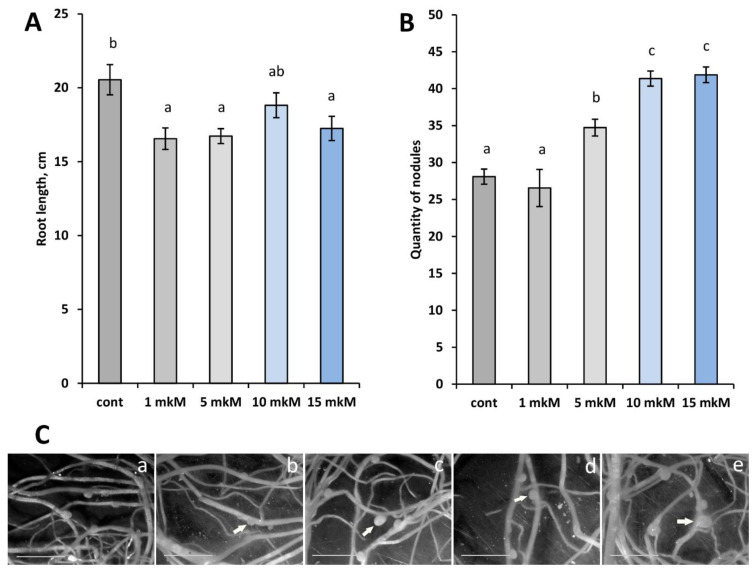
The root length (**A**) and number of nodules (**B**) in *sym33-3* mutant plants non-treated (control, **a**) or treated with increasing concentrations of cytokinin 6-BAP: 1 μM (**b**), 5 μM (**c**), 10 μM (**d**), and 15 μM (**e**). The graphs show the average values of three independent experiments. The error bars represent standard errors of the mean (SEM) of three repeats. The different letters indicate statistically significant differences based on one-way analysis of variance (one-way ANOVA), followed by Tukey post-hoc test. Nodules are indicated by white arrows. A visual increase in the size of the nodules was noted. Scale bar—50 mm.

**Figure 2:**
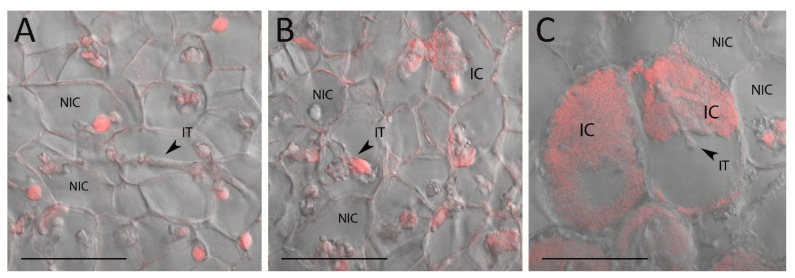
Images of nodule sections of *sym33-3* mutant plants non-treated (**A**) or treated with increasing concentrations of cytokinin 6-BAP such as 5 μM (**B**) and 10 μM (**C**) were obtained using a confocal microscope. Bacteria and plant cell nuclei were stained using propidium iodide. Infected (IC) and non-infected cells (NIC), the infection threads (IT) are marked in the figure. The images are 63X magnified and scaled at 50 µm.

Here, we performed a more detailed morphological and ultrastructural analysis of nodules of pea *sym33-3* mutant plants treated with increasing concentrations of exogenously applied cytokinin 6-BAP in the range from 1 to 15 µM. Similarly to our previous experiments, exogenously applied cytokinin stimulated the total nodule number in mutant (Figure 1); however, we also showed that the nodules in *sym33-3* mutant were increased in size starting with a concentration of 5 to 15 µM (Figure 1C and Figure 2). Since this effect could be related to the later stages of nodule development, we selected several parameters for analysis which were studied in more detail, such as infection thread development and bacterial release, as well as compared with corresponding changes in transcriptome related to the differentiation.

### 2.2. Cytokinin Influences the Growth of Infection Threads and Bacterial Release in Nodules of Sym33-3 Mutant

Serial sections closed to the central part of *sym33-3* nodules in plants treated with increasing concentrations of cytokinin (6-BAP) were used for analysis and compared with control non-treated samples. The total number of infected cells, due to bacterial release as well as the morphology of the growing infection threads, were estimated in the central zone of the nodules that may correspond to the “infection” zone in wild-type nodules (Figure 2).

**Figure 3:**
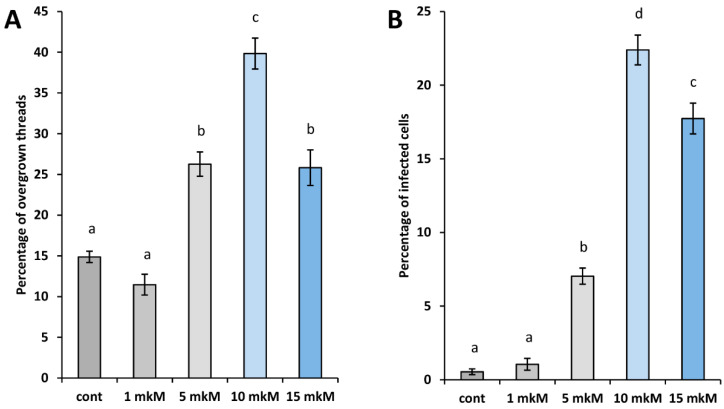
The number of overgrown infection threads (**A**) and percentage of infected cells (**B**) in nodules of *sym33-3* mutant plants non-treated (control) or treated with increasing concentrations of cytokinin 6-BAP: 1 μM, 5 μM, 10 μM, and 15 μM. The graphs represent the average values for 15 sections obtained from 5 nodules in each variant. The error bars represent standard errors of the mean (SEM) of all repeats. The different letters indicate statistically significant differences based on ANOVA, followed by Tukey post-hoc test.

Analysis revealed that the morphology of the infection threads was altered, which often resulted in the appearance of sac-like infection threads in nodules of *sym33-3* mutant plants treated with 5, 10, and 15 μM of 6-BAP compared with control nodules of non-treated *sym33-3* mutant plants (Figure 3A). We defined them as overgrown infection threads. At the same time, the increased number of infected cells, where the bacterial release took place, was observed in nodules of mutant plants treated with 6-BAP starting with 5 μM and reaching the maximum at 10 μM compared with other concentrations in our experimental conditions (Figure 3B). This confirmed that treatment with 10 μM 6-BAP may have the most pronounced stimulating effect on nodule development in the *sym33-3* mutant.

**Figure 4:**
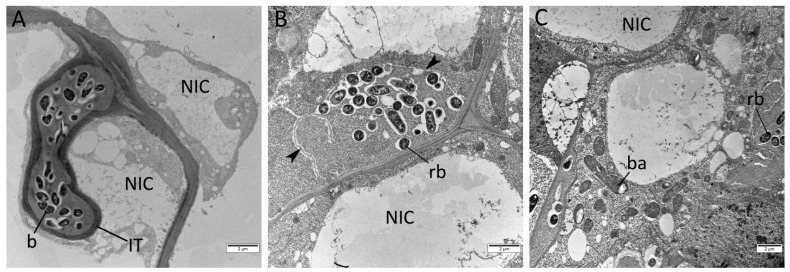
Transmission electron microscopy of nodules of *sym33-3* mutant plants non-treated (**A**) or treated with 10 μM cytokinin 6-BAP (**B**,**C**). There are noticeable differences between nodule cells in non-treated and treated mutant plants, where bacterial release from infection threads took place (**B**,**C**). Image **A** shows an infection thread (IT) with bacteria (b) inside it, surrounded by non-infected cells (NIC). Arrowheads indicate fragmented walls of the infection thread. Images (**B**,**C**) show infected cells with released bacterium (rb) and bacteroides (ba). The figure also shows neighboring non-infected cells (NIC). All figures shown are on a scale of 2 µm.

This phenotype was confirmed by ultrastructural analysis using transmission electron microscopy (TEM). In the nodules of *sym33-3* mutant plants non-treated with cytokinin, bacteria remain inside the “locked” infection threads (Figure 3A) and only very rare events of bacterial release from infection threads may be found, which was in accordance with previous results [30]. In nodule cells with occasional events of bacterial release, the rhizobia were arrested at early stages of development and were not differentiated into bacteroids [37]. However, the cytokinin treatment stimulated the bacterial release and an increased percentage of infected cells may be found in nodules after cytokinin treatment, mainly in response to treatment with 10 µM BAP (Figure 2C and Figure 4B,C). Therefore, a more comprehensive histologic and TEM analysis allowed us to identify the ultrastructural changes in the nodules of the *sym33-3* mutant treated with cytokinin.

### 2.3. Changes in the Expression of Genes Related to Bacterial Release, Accommodation and Subsequent Bacteroid Differentiation Induced by Cytokinin in Sym33 Mutant

To explore the role of possible regulators of infection and bacterial release, we searched for differentially expressed genes related to these processes in pea transcriptome (Table S1) [36]. Transcriptome study in pea *P. sativum* revealed that the Psat7g034920 gene, highly homologous to *MtSYMREM1* encoding remorin protein in *M. truncatula* [38], was significantly induced in nodules of wild-type plants compared with nodules of *sym33-3* mutant and in nodules of *sym33-3* mutant plants treated with 10 µM 6-BAP (Table S1) [36]. It was shown previously that in *M. truncatula,* the MtSYMREM1 remorin may be related to rhizobial infection and bacterial release regulation [38]. Available transcriptome data (RNA-seq) for early stages of symbiosis development 24, 48 h, as well as 3, 4, 5, and 7 days after inoculation (dai) in *M. truncatula* [16] allowed us to perform an additional searching and revealed the strongest stimulation of *MtSYMREM1* gene expression during symbiosis development at later stages. Moreover, predominant localization of the *MtSYMREM1* expression in the infection zone of mature nodules was found in this legume plant (Figure S1) in accordance with previous findings [38]. It suggests that the stimulation of homologous *PsSYMREM1* in response to cytokinin treatment may be important for bacterial release at later stages of nodule differentiation in pea in accordance with previous findings in *Medicago*.

It is known that the regulator of symbiosome differentiation (RSD) is involved in the regulation of later stages of symbiosis development, bacterial accommodation, and promotes symbiosome formation in model legumes [39]. The RSD transcription factor is required for the suppression of defense responses in nodules during bacteria accommodation [40]. This regulator represses the transcription of gene encoding VAMP721a (Vesicle-associated membrane protein 721a) protein, which is important for vesicular transport and secretion. Indeed, analysis of transcriptomes revealed that the level of *PsRSD* (Psat3g136520) gene expression was higher in wild-type pea nodules compared to mutant nodules and up-regulated in response to cytokinin treatment of the *sym33-3* mutant (Table S1) [36].

The important role of the symbiotic gene *MtDNF2* encoding a putative phosphatidylinositol phospholipase C-like protein in the regulation of plant defense responses during bacteria accommodation and stimulation of subsequent bacteroid differentiation was previously shown in *M. truncatula* [41,42]. Additional analysis of our transcriptomic data allowed the identification of the Psat7g125720 gene encoding a putative DNF2 in pea [36]. The expression of this gene was higher in wild-type pea nodules compared with *sym33-3* mutant nodules and showed a significant up-regulation in response to cytokinin treatment (Table S1). In *M. truncatula dnf2 mutant* nodules, the impaired bacteroid differentiation is correlated with the activation of plant defense-like reactions, demonstrating the positive effect of the DNF2 on late stages of nodulation. Therefore, it points towards the existence of a positive link between the stimulating effect of cytokinin on the bacterial accommodation and nodule development, and stimulation of *PsDNF2* in pea nodules.

Small guanosine triphosphate (GTP)-binding proteins from the RAB family, the RAB GTPases, function as signaling regulators and control various aspects of membrane traffic. The pea Psat4g194920 (*PsRab A1*) and Psat6g172840 (PsRab C2) genes were found among differentially up-regulated genes in nodules of wild-type compared to *sym33-3* mutant and up-regulated by cytokinin in the nodules of the mutant (Table S1) [36]. Representatives of these sub-classes, such as *MtRab A1* and *MtRab C1,* were found among up-regulated genes in *M. truncatula* nodules and were shown to be involved in control of the infection process and symbiosome formation [43].

The important role of Nod factor receptors in the regulation of infection process during symbiosis development was shown previously [44,45]. Interestingly, we found the gene *Sym37* (Psat2g024320) encoding one of the receptors to the Nod factor related to the regulation of infection thread growth and development [45] among differentially up-regulated genes in nodules of wild-type compared with *sym33-3* mutant and up-regulated by cytokinin in the nodules of the mutant (Table S1). Therefore, additional activation of genes encoding receptors may be related to bacterial infection and stimulation by cytokinin at these stages.

### 2.4. Analysis of Cytokinin Influence on Regulation of Nodule Morphogenesis

One of the noticeable effects of the influence of exogenously applied cytokinin on nodules in the *sym33-3* mutant was the increasing the size of these structures (Figure 1C). Moreover, the increasing number of infected cells in nodules seems to affect their size (Figure 2C). The growth and differentiation of plant cells result in their enlargement, which is mediated by endoreduplication cycles and related to *CCS52* activation in nodules [23,24]. However, genetic and hormonal networks that regulate the transition to differentiation of nodules are not well characterized in legume plants.

**Figure 5:**
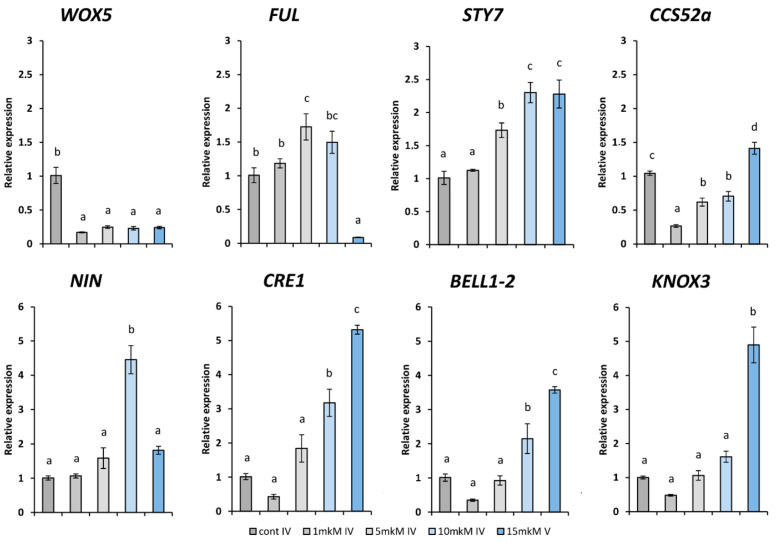
Expression patterns of *WOX5*, *NIN*, *CCS52a*, *CRE1*, *BELL1-2,* and *KNOX3* genes involved in regulation of nodule development as well as newly identified genes encoding STY7 and AGL (FUL) transcription regulators in nodules of *sym33-3* mutant plants non-treated (control) or treated with increasing concentrations of cytokinin 6-BAP: 1 μM, 5 μM, 10 μM, and 15 μM (14 days after inoculation, 14 dai). As a control, the nodules of non-treated *sym33-3* plants were used. The expression was normalized against the constitutively expressed ubiquitin gene. For each gene, the transcript level in nodules of non-treated mutant plants was set to 1 (control), and the level in nodules of cytokinin-treated mutant plants was calculated relative to the control values. Analysis of changes in gene expression was carried out on the basis of three biological repeats. The results of one representative biological repeat were shown out of three, and the standard error of the mean (SEM) of three technical replicates. The different letters indicate statistically significant differences based on one-way analysis of variance (one-way ANOVA), followed by Tukey’s post-hoc test results.

Here, using real-time PCR, we analyzed the expression patterns of *CCS52* and known regulators of nodule morphogenesis in *sym33-3* nodules in response to cytokinin treatment (Figure 5). After treatment with increasing concentrations of exogenously applied cytokinin (1 to 15 µM), we observed a gradual stimulation of the *CCS52A* gene (Psat0s3705g0040) expression as well as the *PsCRE1* gene (Psat7g004720) encoding cytokinin receptor. The expression of the *PsKNOX3* gene (Psat6g028400) encoding transcription factor involved in the regulation of cytokinin biosynthesis through *IPT3, LOG1,* and *LOG2* genes in *M. truncatula* and pea showed similar activation patterns [7,46]. In addition, the *PsNIN* gene (Psat2g001120) and *PsBELL1-2* (Psat4g090560) encoding transcription factors showed a concentration-dependent activation of expression in accordance with previous findings about cytokinin-stimulated expression of these genes in other legumes [12,47,48].

The cytokinin-mediated up-regulation of *CCS52* was in line with the data in *Arabidopsis*, where cytokinin, through the cytokinin-activated B-type response regulator ARR2, directly up-regulates the expression of the *CCS52A1* gene encoding an activator of an ubiquitin ligase complex (the anaphase-promoting complex/cyclosome) and mediating degradation of cell cycle regulators [49,50]. In *Arabidopsis,* this regulator co-ordinates root growth by promoting endoreduplication and restricting cell proliferation in the root meristem. Therefore, such regulation by cytokinin may be related to later stages of nodule development.

Previous studies in legume plants have shown that the transcription factor EFD is required for the formation of functional nodules and essential for nodule differentiation [51]. The enlargement in nodule size in the *sym33-3* mutant treated with exogenous cytokinin can be also associated with the stimulation of the Psat7g259160 gene encoding PsEFD, which we previously described in transcriptomes as significantly induced in nodules of wild-type plants compared with nodules of the *sym33-3* mutant and in nodules of *sym33-3* mutant plants treated with cytokinin (Table S1) [36]. The activity of this gene is associated with the differentiation of the zone of infection in model legumes [51], which is consistent with our results.

**Figure 6:**
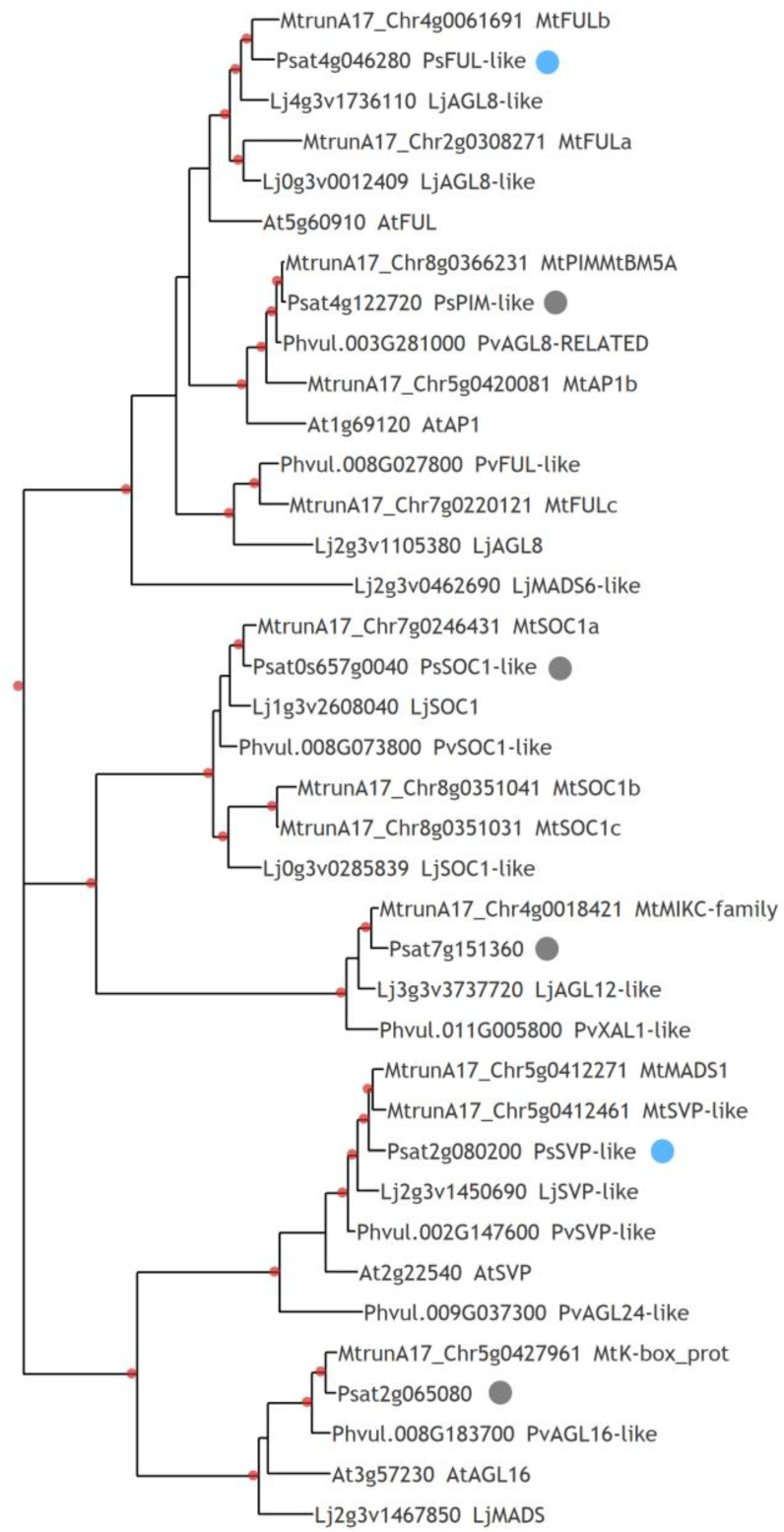
Phylogenetic tree constructed by the maximum likelihood method based on the amino acid sequences of the AGL family genes in *P. sativum*, *P. vulgaris L. japonicus, A. thaliana,* and *M. truncatula*. Red dots indicate over 0.7 support based on 1000 iterations of the bootstrap method. Blue dots indicate genes showing increased expression upon cytokinin treatment of *sym33-3* mutant plants. Gray dots indicate genes whose expression in *sym33-3* mutant nodules is lower than in wild-type pea nodules.

We also estimated the effect of exogenously applied cytokinin in the range of 1 to 15 µM for newly identified genes related to growth and development regulation in plants, which were previously found among differentially expressed genes as up-regulated in wild-type nodules compared with *sym33-3* mutant nodules as stimulated in *sym33-3* mutant nodules in response to cytokinin treatment (Table S1) [36]. The MADS-domain/AGAMOUS-LIKE (AGL) transcription factors belong to a large family of regulators involved in the development of plant organs. The stimulating effect of exogenously applied cytokinin in the range of 1 to 15 µM was shown for the Psat4g046280 gene encoding MADS-domain/AGL transcription factor in *sym33-3* pea nodules (Figure 5). This gene seems to be a homologue of the *FRUITFULL-like b* (*MtFULb*) gene (83.78 % of identity) in *M. truncatula* [52], which plays an important role in the development of inflorescences, including the control of flowering time and inflorescence meristem identity. Therefore, the homologous gene Psat4g046280 in pea was called *PsFUL* (Figure 5). Interestingly, several members of the MADS-domain/AGL transcription factor family were recently found to be differentially regulated during *Phaseolus vulgaris*–rhizobia interaction and related to rhizobial infection and nodule development [53]. Here, we searched for additional MADS-domain/AGL transcription factor homologues in the pea genome v. 1 https://urgi.versailles.inra.fr/Species/Pisum/Pea-Genome-project accessed on 1 January 2023, using BLAST sequence analysis against recently identified genes of the AGL family in *P. vulgaris* related to nodule development [53]. The related genes from *M. truncatula* were also used for searching, which allowed us to identify a number of pea homologues (Table S2) for use in a phylogenetic analysis (Figure 6).

This resulted in the identification of an additional Psat2g080200 gene encoding MADS-domain/AGL transcription factor, which is the closest homologue of SHORT VEGETATIVE PHASE-like (PvSVP-like, 87.90% of identity) in *P. vulgaris,* and *MtMADS1* (90.45% of identity), and *MtSVP-like* (88.54%) in *M. truncatula*. The gene *PsSVP*-*like* (Psat2g080200) was highly expressed in wild-type nodules compared with nodules of the *sym33-3* mutant and showed to be stimulated in response to cytokinin treatment [36]. In addition, using the transcriptomic data for *M. truncatula* at 24 and 48 h, as well as 3, 4, 5, and 7 dai [16] (Figure S2), the transcription levels of homologous genes of MADS-domain/AGL family were estimated as well as their localization in mature nodules (Figure S3). Analysis has shown the highest level of activation in response to inoculation for the *MtMADS1* (the closest homologue of Psat2g080200 (*PsSVP-like*) (Figure S3A) up to 7 dai, and its high expression level in the nodules (Figure S3B). The similar patterns of MADS-domain/AGL family gene expression in *P. vulgaris* and *M. truncatula* demonstrate their important role in mature nodule development. Therefore, it indicates a complex network of MADS-domain/AGL transcription factors in the regulation of the later stages of nodule development triggered by cytokinin.

**Figure 7:**
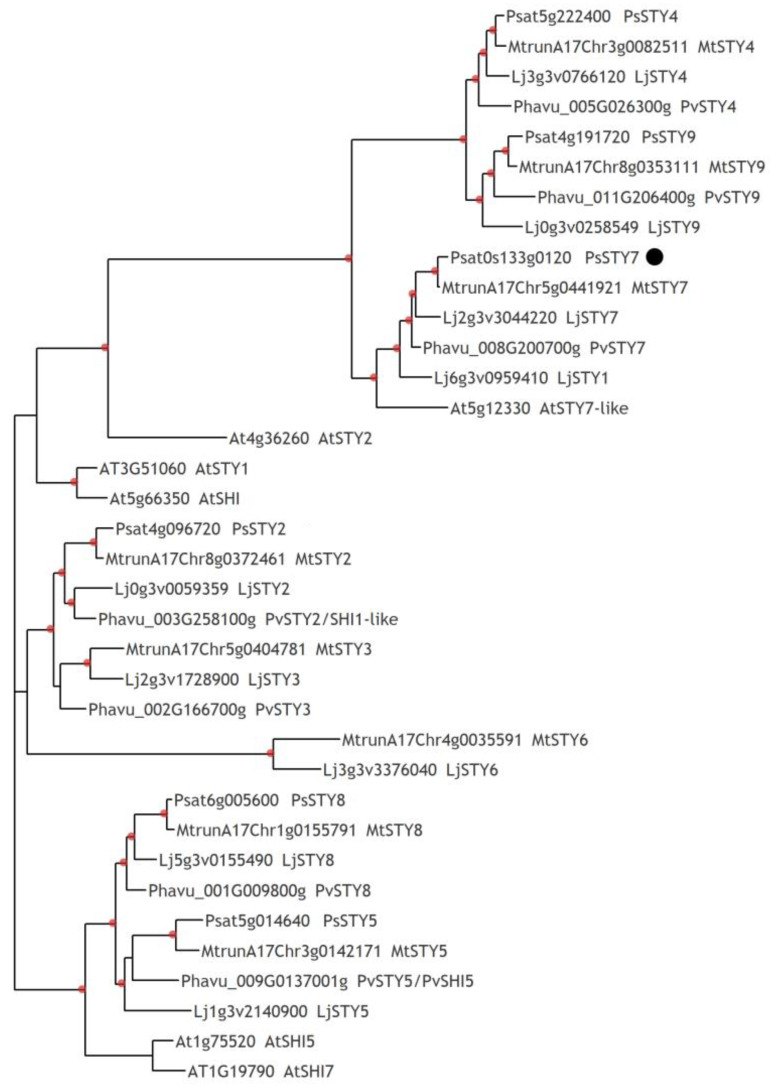
Phylogenetic tree constructed by the maximum likelihood method based on the amino acid sequences of the STY family genes in model legume plants *L. japonicus, P. vulgaris, A. thaliana,* and *M. truncatula*, as well as in *P. sativum*. Red dots indicate over 0.7 support based on 1000 iterations of the bootstrap method. The black dot indicates the gene of interest.

The important role of the *SHORT INTERNODE*/*STYLISH (SHI/STY)* gene family was recently shown in the regulation of nodule development in legume *L. japonicus* [54,55]. Previously, among differentially expressed genes, we found the Psat0s133g0120 gene, the closest homologue of *LjSTY1* and *LjSTY7* genes from *L. japonicus* [36]. Searching in the Phytozome database v. 13 (https://phytozome.jgi.doe.gov/ accessed on 1 January 2023) for *M. truncatula* resulted in identification of nine *MtSTY* genes. Based on BLASTP sequence analysis, we found a set of the *SHI/STY* gene family homologues in pea (Table S3) and performed a phylogenetic analysis (Figure 7). As a result, we confirmed that the Psat0s133g0120 gene as the *PsSTY7*, the closest homologue of *MtSTY7* and *LjSTY1*, *LjSTY7* in *M. truncatula* and *L. japonicus*, respectively. Here, the stimulating effect of exogenously applied cytokinin in the range of 1 to 15 µM was also revealed for this *PsSTY7* gene (Figure 5), in accordance with our previous data [36].

Subsequent analysis of the expression levels of identified *MtSTY1-9* genes during nodule development in *M. truncatula* (from 24 h up to 7 dai) [16] showed stimulation of all of them. However, the highest level of expression at later nodulation stages as well as in nodules compared to roots was shown for *MtSTY7* (Figure S4A). Interestingly, the localization of *MtSTY7* gene expression was prevailing in the meristem (Figure S4B). The *SHI/STY* genes are known to induce the expression of auxin biosynthesis genes such as *YUCCA* [56,57], and the *MtSTY7* gene localization may likely be related to the maintenance of auxin maximum in the apical meristem in mature nodules. Generally, the genes of the SHI/STY family affect growth and development and may be important for the specification of tissue identity and differentiation of generative organs [58] and carpels [59], and specification of the apical meristems [55,60]. The SHI/STY can perform a similar function in various plants and mosses [57], indicating a conserved role for SHI/STY in various tissues. The stimulation of *PsSTY7* by cytokinin may be important for subsequent specification of nodule tissue identity. Further experiments will be helpful to reveal possible molecular mechanisms of its function.

Cytokinin was shown to negatively affect the accumulation of PIN auxin efflux carriers during main root and lateral root development [49,61] as well as during nodule primordium formation [12,62]. To estimate the role of auxin at the later stages of nodulation and in response to cytokinin treatment, we found a number of *PIN* genes encoding PINOID (PIN) auxin efflux transporters in pea. Analysis of the pea genome allowed us to find at least 13 homologues of the *PIN* genes during searching against 11 homologues in *M. truncatula* (Table S4). It was shown that the level of *PsPIN1* (Psat7g127400), *PsPIN5* (Psat7g003040), and *PsPIN7* (Psat4g014160) gene expression was significantly lower in wild-type nodules compared with *sym33-3* mutant nodules [36]. This suggests that during the development of effective nodules in wild-type plants, the expression of these *PIN* genes is suppressed, while this effect was abolished in the *sym33-3* (*ipd3/cyclops)* mutant. Therefore, it seems that the cytokinin accumulation correlates with the down-regulation of some genes encoding PIN auxin efflux carriers not only at nodule primordium formation, but also in mature nodules.

At the same time, among differentially expressed genes, one pea *PIN* gene, the *PsPIN2* (Psat4g014200), showed a significant up-regulation in nodules of the *sym33-3* mutant treated with exogenous cytokinin [36]. Using available data for *M. truncatula* at 24 and 48 h, as well as 3, 4, 5, and 7 days after inoculation (dai) [16], we also analyzed the *MtPIN1-MtPIN11* gene expression in *M. truncatula* and found four up-regulated genes upon inoculation with rhizobia: the *MtPIN1, MtPIN2, MtPIN6,* and *MtPIN10* (Figure S5). However, in mature nodules, their predominant localization was found in the meristem zone. Therefore, these PIN auxin efflux carriers may be related to supporting the auxin level in the meristem of mature nodules in pea and *M. truncatula*. Therefore, the role of PIN auxin efflux carriers may be different, depending on the localization and stage of nodule development. The expression of some of them is suppressed in mature nodules, but others may be related to the support of auxin maximum in the meristems.

Besides the regulators of the auxin redistribution such as PINs, some genes involved in the control of auxin biosynthesis such as *YUCCA* were found among those differentially expressed in our transcriptomes. One of the genes, the *YUCCA6* (Psat5g023680), showed decreased expression in the nodules of wild-type plants compared with nodules of the *sym33-3* mutant. This was in line with situation for some *PIN* genes, having a higher level of expression in the nodules of the *sym33-3* pea mutant. At the same time, several genes such as *PsYUCCA1* (Psat6g030600) and *PsYUCCA10* (Psat5g146760) were up-regulated by cytokinin in the nodules of the *sym33-3* mutant, which we believe to be positive regulators of meristem development.

### 2.5. Conclusions

This study provides knowledge about how cytokinin influences the nodule morphogenesis in pea *P. sativum* plants, using the pea *sym33-3* mutant (*ipd3/cyclops*) and exogenous applications of 6-BAP (cytokinin). Cytokinin treatment was shown to stimulate bacterial release and increase the percentage of infected cells in *sym33-3* mutant nodules. To explore the role of possible regulators of nodule differentiation, we performed searching in pea transcriptome. Transcriptome analysis gave a new insight on a program of symbiotic nodule development at late stages in pea *P. sativum* and revealed a network of regulators involved in this process.

## 3. Materials and Methods

### 3.1. Bacterial Strains and Inoculation

Plants were inoculated with the *Rhizobium leguminosarum biovar viciae* CIAM 3841 strain, which was cultured at 28 °C on yeast extract mannitol agar medium (YEM) [63] supplemented with 0.6 mg/mL streptomycin. For preparation of inoculum, bacteria were incubated in liquid B—medium [64] until the required density was obtained. The optical density of the suspension at 600 nm (OD_600_) was adjusted to 0.8–1.0.

### 3.2. Plant Material and Growth Conditions

Wild-type cv. SGE and mutant SGEFix^—^2 (*sym33-3*) of *Pisum sativum L.* were taken from collection of ARRIAM (St. Petersburg, Russia). The seeds were sterilized with sulfuric acid for 10 min, washed 5 times with sterile distilled water and left to swell in water with moderate stirring for 40–60 min, and then the seeds were transferred to plates with 1% aqueous agar and stratified at +4 °C overnight for uniform germination. The seeds were then germinated at room temperature in the dark for 5–6 days. After germination, the seedlings were transferred to vermiculite pots soaked in Jensen’s medium and grown in a climate chamber (Binder, Tuttlingen, Germany,) at 21 °C with cycles of 16 h light/8 h dark and 64% humidity. Pea seedlings were inoculated with 2 mL of *R. leguminosarum bv. Viciae* CIAM 3841 per seedling. After 5 days, the plants were treated with 6-BAP with several final concentrations such as 1 µM, 5 µM, 10 µM, and 15 µM and a volume of 50 mL per pot (diluted with sterile distilled water). The treatment was carried out every day, the control plants were watered in the same volume with sterile distilled water. Nodules were collected after 14 days for cytological and gene expression analysis.

### 3.3. Material Fixation and Staining of Sections

The nodules were fixed on ice in a freshly prepared solution of 3% paraformaldehyde (Sigma-Aldrich, Burlington, MA, USA) in MTSB buffer of 1/3 strength (50 mM PIPES (pH 6.9) (Amresco, Boise, ID, USA); 5 mM MgSO_4_x7H_2_O; 5 mM ethylene glycol-bis (β-aminoethyl ether) -N, N, N′, N′-tetraacetic acid (Sigma-Aldrich, Burlington, MA, USA) with the addition of 0.25% glutaraldehyde (solution Grade I, 25% in H_2_O. Sigma-Aldrich, Burlington, MA, USA), 0.3% Twin-20 (Amresco, Boise, ID, USA), 0.3% Triton-X-100 (Amresco, Boise, ID, USA). For optimal penetration of the fixative, the air from the tissue was evacuated 3 times for 7 min at 0.9 bar using a ME 1 vacuum pump (Vacuubrand, Wertheim, Germany) and left overnight at +4 °C. Then, the material was washed with PBS buffer (0.137 M NaCl, 0.0027 KCl, 0.01 M Na_2_HPO_4_, 0.0018 M KH_2_PO_4_, pH 7.4). Fixed nodules were embedded in 2.5% agarose (Agarose, 1/4 MTSB) and sectioned on a vibrotome (TED PELLA, Inc., Redding, CA, USA). Sections were then freed from agarose and stained with propidium iodide.

### 3.4. Transmission Electron Microscopy

The nodules were fixed in solution of 2.5 % (*v*/*v*) glutaraldehyde (solution Grade I, 25% in H_2_O. Sigma-Aldrich, Burlington, MA, USA) in PBS buffer overnight, post-fixed in 1.5% OsO_4_ (EMS, Hatfield, PA, USA) in the same buffer, dehydrated in ethanol series and acetone and embedded in Epon EmBed812 (EMS, Hatfield, PA, USA). Ultrathin sections were made using ultratome EM UC7 (Leica, Vienna, Austria) and stained on grids with 2% uranyl acetate (SPI, West Chester, PA, USA) and Reynolds’ lead citrate [65]. Sections were photographed in transmission electron microscope JEM-1400 (Jeol, Tokyo, Japan) equipped with side camera Veleta (Olympus, Tokyo, Japan) at 80 kV.

### 3.5. Isolation of RNA and Quantitative Reverse Transcription PCR (qRT-PCR)

The total RNA was isolated from the nodules of inoculated or treated with cytokinin plants using the PureZol reagent (Bio-Rad Laboratories, Philadelphia, PA, USA) according to the manufacturer’s protocol. To remove genomic DNA, DNAse I treatment (NEB, Ipswich, MA, USA) was used. Complementary DNA was prepared from 30–45 µg of RNA using RevertAid H Minus Reverse Transcriptase (Thermo Fisher Scientific, Waltham, MA, USA) using oligo(dT) primers and RNase Inhibitor (NEB, Ipswich, MA, USA). Quantitative reverse transcription PCR (qRT-PCR) was performed using the CFX96 real-time system (Bio-Rad Laboratories, Philadelphia, PA, USA) and 5X qPCRmix-HS SYBR (Evrogen, Moscow, Russia). All primer pairs (Table S5) were designed using the Vector NTI program and were manufactured by Evrogen (www.evrogen.com accessed on 1 January 2023). mRNA levels were normalized to ubiquitin and values were calculated as ratios to expression levels of untreated roots.

### 3.6. Searching Homologues of Known Genes and Their Analysis

We used the BLASTP algorithm to search for homologues of genes in pea *P. sativum* genome v 1.0. Functional gene annotation in pea was done based on the highest level of similarity to genes from other plants. *M. truncatula* A17 r5.0 genome version [66] was used for gene annotation in *M. truncatula.* The expression levels of the genes in *M. truncatula* at different stages upon inoculation as well as in various nodule zones were presented based on a reanalysis of RNA-Seq data from the PRJNA552042 project [16] using the *M. truncatula* r5.0 genome as a reference. Homologous sequences were found in *Arabidopsis thaliana* [67] and *Phaseolus vulgaris* [68] genomes as well as *Lotus japonicus* [69]. This analysis was carried out in R studio using a ready-made script [48].

### 3.7. Phylogenetic Analysis

Amino acid sequences were aligned using the ClustalW algoritm. Phylogenetic trees for the AGL and STY gene families were constructed using maximum-likelihood algorithms and the JTT matrix model [70], and statistical support for the branch topology was calculated from 1000 bootstrap replicates [71]. The source trees for the heuristic search were automatically generated by applying the Neighbor-Join and BioNJ algorithms to a matrix of pairwise distances estimated using the JTT model and then selecting a topology with a higher log-likelihood value. The analysis involved 38 amino acid sequences of representatives of the AGL family and 36 amino acid sequences of STY proteins. In total, the final dataset contained 276 positions for AGL and 493 positions. An evolutionary analysis was carried out in R Studio.

### 3.8. Statistical Analysis

One-way analysis of variance (ANOVA) was used to check the differences in root length and number of nodules. Data of three independent biological repeats (4–5 plants per replicate) were used for analysis. Tukey post-hoc test was performed between all groups to determine the differences within the groups.

For cell counts in each variant, at least fifteen slices of nodules from each of three different biological repeats were used. The percentage of overgrown infection threads was compared in X and Y, and to assess statistical significance, ANOVA with Tukey post-hoc test was used. Analysis of changes in gene expression was carried out on the basis of 3 biological repeats and included 4–5 plants for each control and treated variants in 1 repeat. The results of one representative biological repeat were shown out of three, and the standard error of the mean (SEM) of three technical replicates. The threshold cycle (Ct) values were calculated using the Bio-Rad CFX Manager 1.6 program and analyzed using the 2^−ΔΔCt^ method. Standard one-way ANOVA analysis and Tukey post-hoc test were performed.

## Data Availability

The authors declare that all data supporting the findings of this study are available within this article and its Supplementary Information files.

## Supplementary Materials

The following materials are available online at https://www.mdpi.com/article/10.3390/plants12030657/s1. Figure S1. (A) Expression level of *M. truncatula MtSYMREM1* gene based on re-analysis of RNA-Seq data from PRJNA552042 project [16] using *M. truncatula* genome v.5 as a reference. Bars represent means of counts per million values (CPM) based on 4–6 replicates. (B) Expression level of *M. truncatula MtSYMREM1* genes in the different nodule zones based on re-analysis. (C) Graphic illustration of data for *M. truncatula MtSYMREM1* genes expression in the roots and nodules based on re-analysis of RNA-Seq data. Zone I (ZI)—bacteria-free meristematic zone; zone II (ZIIp)—the proximal part of the infection zone; zone II (ZIId)—the distal part of the infection zone; zone III (ZIII)—symbiosomes, which consist of differentiated bacteroids; interzone (IZ)—several layers of cells wide between ZII and ZIII. Figure S2. Expression levels of *M. truncatula* genes encoding MADS-domain/AGL transcription factors at the early stages of nodulation based on re-analysis of RNA-Seq data from PRJNA552042 project using *M. truncatula* genome v.5 as a reference. Figure S3. (A) Graphic illustration of data for *M. truncatula MADS-domain/AGL* genes expression in the roots and nodules based on re-analysis of RNA-Seq data. (B) Expression level of *M. truncatula MADS-domain/AGL* genes in the different nodule zones. Zone I (ZI)—bacteria free meristematic region; zone II (ZIIp)—the proximal part of the infection zone; zone II (ZIId)—the distal part of the infection zone; zone III (ZIII)—symbiosomes, which consist of differentiated bacteroids; the interzone (IZ)—a few cell layers wide between ZII and ZIII. Figure S4. Expression levels of *M. truncatula SHI/STY* genes based on re-analysis of RNA-Seq data from PRJNA552042 project [16] using *M. truncatula* genome v.5 as a reference. (B) Expression level of *M. truncatula SHI/STY* genes in the different nodule zones. Zone I (ZI)—bacteria free meristematic region; zone II (ZIIp)—the proximal part of the infection zone; zone II (ZIId)—the distal part of the infection zone; zone III (ZIII)—symbiosomes, which consist of differentiated bacteroids; interzone (IZ)—several layers of cells between ZII and ZIII. Figure S5. (A) Expression levels of *M. truncatula PIN* genes based on re-analysis of RNA-Seq data from PRJNA552042 project [16] using *M. truncatula* genome v.5 as a reference. (B) Expression level of *M. truncatula PIN* genes in the different nodule zones. Zone I (ZI)—bacteria-free meristematic zone; zone II (ZIIp)—the proximal part of the infection zone; zone II (ZIId)—the distal part of the infection zone; zone III (ZIII)—symbiosomes, which consist of differentiated bacteroids; interzone (IZ)—several layers of cells between ZII and ZIII. Table S1. List of up-regulated expressed genes in pea nodules of cv. SGE wild-type and SGEFix^-^-2 (*sym33*) mutant as well as in pea nodules of SGEFix^-^-2 (*sym33*) mutant plants untreated or treated with cytokinin based on Venna diagram using cutoff threshold equal log2 fold change value > 2 and *p* adjusted value < 0.05. Table S2. The IDs for MADS-domain/AGAMOUS-LIKE (AGL) gene family and their homologues in *Medicago truncatula*, *Phaseolus vulgaris* and *Pisum sativum*. Table S3. The IDs for SHORT INTERNODE/STYLISH (SHI/STY) gene family and their homologues in legume species such as *Lotus japonicus, Medicago truncatula* and *Pisum sativum*. Table S4. Identified homologues of *PIN* genes in *Medicago truncatula* and *Pisum sativum.* Table S5. List of primers.
