# Supplementary material for: Exogenously Applied Cytokinin Altered the Bacterial Release and Subsequent Stages of Nodule Development in Pea Ipd3/Cyclops Mutant
Source: Plants (Basel). 2023 Feb 2;12(3):657. doi: 10.3390/plants12030657 (PMC9921755; doi:10.3390/plants12030657)
Supplement: Supplementary file 1 [file plants-12-00657-s001.zip › Table S2_corr.pdf]

Supplementary table S2. *MADS-domain/ AGAMOUS-LIKE (AGL)* gene family and their homologues in *Medicago truncatula*, *Phaseolus vulgaris* and *Pisum sativum*.

Gene name	Accession №	Organism	References
AtFUL	At5g60910	*Arabidopsis thaliana*	Chu T et al., 2010
AtAP1	At1g69120	*Arabidopsis thaliana*	Monniaux et al., 2018
AtSVP	At2g22540	*Arabidopsis thaliana*	Liu et al., 2018
AtAGL16	At3g57230	*Arabidopsis thaliana*	Kutter C et al., 2007
LjMADS	Lj2g3v1467850	*Lotus japonicus*	-
LjMADS6-like	Lj2g3v0462690	*Lotus japonicus*	-
LjSVP-like	Lj2g3v1450690	*Lotus japonicus*	-
LjSOC1	Lj1g3v2608040	*Lotus japonicus*	Kou et al., 2022
LjAGL8	Lj2g3v1105380	*Lotus japonicus*	-
LjAGL8-like	Lj0g3v0012409	*Lotus japonicus*	-
LjAGL8-like	Lj4g3v1736110	*Lotus japonicus*	-
LjAGL12-like	Lj3g3v3737720	*Lotus japonicus*	-
MtMADS1	MtrunA17_Chr5g0412271	*Medicago truncatula*	Moreau et al., 2011
MtFULa	MtrunA17_Chr2g0308271	*Medicago truncatula*	Jaudal et al., 2015
MtFULb	MtrunA17_Chr4g0061691	*Medicago truncatula*	Jaudal et al., 2015
MtFULc	MtrunA17_Chr7g0220121	*Medicago truncatula*	Cheng et al., 2018
MtSOC1a	MtrunA17_Chr7g0246431	*Medicago truncatula*	Cheng et al., 2021
MtSOC1b	MtrunA17_Chr8g0351041	*Medicago truncatula*	Cheng et al., 2021
MtSOC1c	MtrunA17_Chr8g0351031	*Medicago truncatula*	Cheng et al., 2021
MtAP1b	MtrunA17_Chr5g0420081	*Medicago truncatula*	Zhu et al., 2018
MtSVP-like	MtrunA17_Chr5g0412461	*Medicago truncatula*	Cheng et al., 2021
MtMIKC	MtrunA17_Chr4g0018421	*Medicago truncatula*	-
MtK-box	MtrunA17_Chr5g0427961	*Medicago truncatula*	-
MtPIM, MtBM5A	MtrunA17_Chr8g0366231	*Medicago truncatula*	Jaudal et al., 2015 Benlloch et al., 2006
PsFUL-like	Psat4g046280	*Pisum sativum*	Rudaia et al., 2021
PsSVP-like	Psat2g080200	*Pisum sativum*	-
PsPIM-like	Psat4g122720	*Pisum sativum*	-
-	Psat7g151360	*Pisum sativum*	Kusakin et al., 2021
-	Psat2g065080	*Pisum sativum*	-
-	Psat0s657g0040.1	*Pisum sativum*	-

PvFUL-like	Phvul.008G027800	*Phaseolus vulgaris*	Ayra et al., 2021
PvSVP-like	Phvul.002G147600	*Phaseolus vulgaris*	Ayra et al., 2021
PvSOC1-like	Phvul.008G073800	*Phaseolus vulgaris*	Ayra et al., 2021
PvAGL8-like	Phvul.003G281000	*Phaseolus vulgaris*	
PvAGL16-like	Phvul.008G183700	*Phaseolus vulgaris*	Ayra et al., 2021
PvAGL24-like	Phvul.009G037300	*Phaseolus vulgaris*	Ayra et al., 2021
PvXAL1-like	Phvul.011G005800	*Phaseolus vulgaris*	Ayra et al., 2021
