# Supplementary material for: Exogenously Applied Cytokinin Altered the Bacterial Release and Subsequent Stages of Nodule Development in Pea Ipd3/Cyclops Mutant
Source: Plants (Basel). 2023 Feb 2;12(3):657. doi: 10.3390/plants12030657 (PMC9921755; doi:10.3390/plants12030657)
Supplement: Supplementary file 1 [file plants-12-00657-s001.zip › Table S3_corr.pdf]

Supplementary table S3. *SHORT INTERNODE/STYLISH (SHI/STY)* gene family and their homologues in legume species such as *Lotus japonicus, Medicago truncatula* and *Pisum sativum*.

Gene name	Accession number	Organism	Homology for *PsSTY7*	References
AtSHI	*At5g66350*	*Arabidopsis thaliana*	37.65	Fridborg et al., 2001
AtSTY1	*At3g51060*	*Arabidopsis thaliana*	35.25	Kuusk et al., 2002
AtSTY2	*At4g36260*	*Arabidopsis thaliana*	38.67	Kuusk et al., 2002
AtSTY7-like	*At5g12330*	*Arabidopsis thaliana*	54.05	Kuusk et al., 2006
AtSHI5	*At1g75520*	*Arabidopsis thaliana*	33.09	Kuusk et al., 2006
AtSHI7	*AT1G19790*	*Arabidopsis thaliana*	33.58	Kim et al., 2010
LjSTY1	Lj6g3v0959410	*Lotus japonicus*	66.90	Shrestha et al., 2021
LjSTY2	Lj0g3v0059359	*Lotus japonicus*	38.87	Shrestha et al., 2021
LjSTY3	Lj2g3v1728900	*Lotus japonicus*	35.80	Shrestha et al., 2021
LjSTY4	Lj3g3v0766120	*Lotus japonicus*	39.67	Shrestha et al., 2021
LjSTY5	Lj1g3v2140900	*Lotus japonicus*	38.26	Shrestha et al., 2021
LjSTY6	Lj3g3v3376040	*Lotus japonicus*	34.67	Shrestha et al., 2021
LjSTY7	Lj2g3v3044220	*Lotus japonicus*	70.90	Shrestha et al., 2021
LjSTY8	Lj5g3v0155490	*Lotus japonicus*	34.10	Shrestha et al., 2021
LjSTY9	Lj0g3v0258549	*Lotus japonicus*	42.56	Shrestha et al., 2021
MtSTY2	MtrunA17Chr8g0372461	*Medicago truncatula*	37.70	Shrestha et al., 2021
MtSTY3	MtrunA17Chr5g0404781	*Medicago truncatula*	33.73	Shrestha et al., 2021
MtSTY4	MtrunA17Chr3g0082511	*Medicago truncatula*	43.40	Shrestha et al., 2021
MtSTY5	MtrunA17Chr3g0142171	*Medicago truncatula*	35.27	Shrestha et al., 2021
MtSTY6	MtrunA17Chr4g0035591	*Medicago truncatula*	34.32	Shrestha et al., 2021
MtSTY7	MtrunA17Chr5g0441921	*Medicago truncatula*	88.86	Shrestha et al., 2021
MtSTY8	MtrunA17Chr1g0155791	*Medicago truncatula*	33.58	Shrestha et al., 2021
MtSTY9	MtrunA17Chr8g0353111	*Medicago truncatula*	40.96	Shrestha et al., 2021
PvSTY2	Phavu_003G258100g	*Phaseolus vulgaris*	37.97	-
PvSTY3	Phavu_002G166700g	*Phaseolus vulgaris*	36.61	-
PvSTY4	Phavu_005G026300g	*Phaseolus vulgaris*	41.28	-
PvSTY5	Phavu_009G0137001g	*Phaseolus vulgaris*	33.74	-
PvSTY7	Phavu_008G200700g	*Phaseolus vulgaris*	72.98	-
PvSTY8	Phavu_001G009800g	*Phaseolus vulgaris*	33.21	-

PvSTY9	Phavu_011G206400g		39.72	
PsSTY2	Psat4g096720.1	*Pisum sativum*	37.35	-
PsSTY4	Psat5g222400.1	*Pisum sativum*	43.00	-
PsSTY5	Psat5g014640.1	*Pisum sativum*	32.79	-
PsSTY7	Psat0s133g0120.1	*Pisum sativum*	-	Rudaia et al., 2021
PsSTY8	Psat6g005600.1	*Pisum sativum*	31.01	-
PsSTY9	Psat4g191720.1	*Pisum sativum*	40.62	-
