# Supplementary material for: Exogenously Applied Cytokinin Altered the Bacterial Release and Subsequent Stages of Nodule Development in Pea Ipd3/Cyclops Mutant
Source: Plants (Basel). 2023 Feb 2;12(3):657. doi: 10.3390/plants12030657 (PMC9921755; doi:10.3390/plants12030657)
Supplement: Supplementary file 1 [file plants-12-00657-s001.zip › Table S4_corr.pdf]

Supplementary table S4. Identified homologues of *PIN* genes in *Medicago truncatula* and *Pisum sativum*

Gene	Accession number (*Medicago truncatula*)	Accession number (*Pisum sativum*)
PIN1	Medtr4g084870	Psat7g127400
PIN2	Medtr4g127100	Psat4g014160
PIN3	Medtr1g030890	Psat6g043160
PIN4	Medtr6g069510	Psat1g032680
PIN5	Medtr8g107360	Psat7g003040
PIN6	Medtr1g029190	Psat0s502g0040
PIN7	Medtr4g127090	Psat4g014200
PIN8	Medtr7g009370	Psat3g195480
PIN9	Medtr7g079720	Psat3g103640
PIN10	Medtr7g089360	Psat3g080160
PIN11	Medtr6g011400	Psat0s564g0040
PIN12	-	Psat0s1191g0040
PIN13	-	Psat4g014080
