# Supplementary material for: Exogenously Applied Cytokinin Altered the Bacterial Release and Subsequent Stages of Nodule Development in Pea Ipd3/Cyclops Mutant
Source: Plants (Basel). 2023 Feb 2;12(3):657. doi: 10.3390/plants12030657 (PMC9921755; doi:10.3390/plants12030657)
Supplement: Supplementary file 1 [file plants-12-00657-s001.zip › Table S5_corr.pdf]

Supplementary table S5. List of primers used in this study

PsUbiq For	ATG CAG ATY TTT GTG AAG AC
PsUbiq For	ACC ACC ACG RAG ACG GAG
PsSTY For	AAC TAC TTC TCA CAC TTC CA
PsSTY Rev	GCA TAC TCG TCT TTT CCA TC
PsFUL-like For	GCT TTT TGA ATA CTC TAG CGA
PsFUL-like Rev	GAG ACT TAA CCC ATC CAA CT
PsNIN For	ATG CCT TCT TCT GCT TCC GAT G
PsNIN Rev	CTT ATG GTC TTC TCC GCC TTG GT
PsCRE1 For	CCA ACG GGA TTA TGC TCA GAC AG
PsCRE1 Rev	CTC CCA GGA TCC CCC ATA ACA ATA
PsBEL1-2 For	CTC ACG GCG CCT CTC CTG
PsBEL1-2 Rev	TGA AAT ATG CTG CTG CTG CTA CTG
PsKNOX3 For	CAA AGC TTA ACA GGA GTT TCA CC
PsKNOX3 Rev	CTG TAG GTA TAA GAG GGC CAA ATC
PsCCS52a For	CAA GAC GGA GAC TCG GCA GT
PsCCS52a Rev	GCC CAG CCA ACA GAA CAA AC
PsWOX5 For	GGT TTC AAA ATC ATA AGG CTA GGG A
PsWOX5 Rev	TCA ACC GCA AGT CTA ATG GTG GAT G
