# Supplementary figures and images for: Exogenously Applied Cytokinin Altered the Bacterial Release and Subsequent Stages of Nodule Development in Pea Ipd3/Cyclops Mutant
Source: Plants (Basel). 2023 Feb 2;12(3):657. doi: 10.3390/plants12030657 (PMC9921755; doi:10.3390/plants12030657)

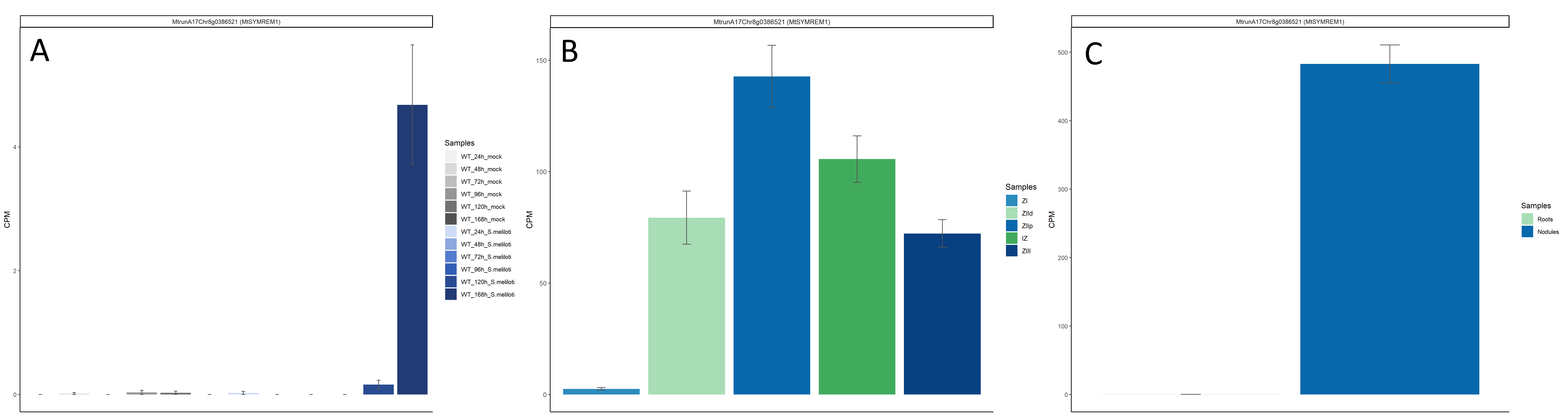

Supplement: Supplementary file 1 [file plants-12-00657-s001.zip › Figure S1.tiff]

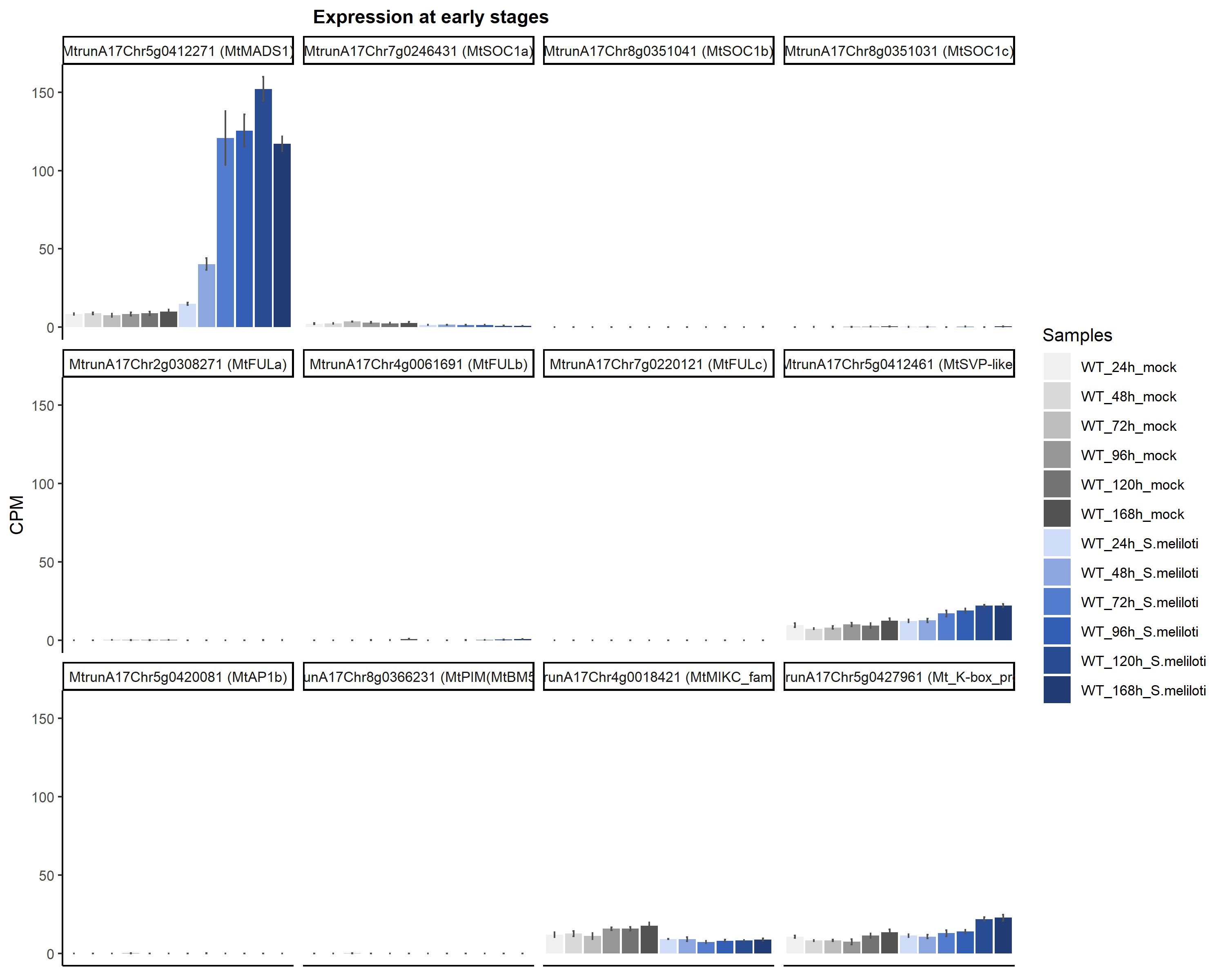

Supplement: Supplementary file 1 [file plants-12-00657-s001.zip › Figure S2.png]

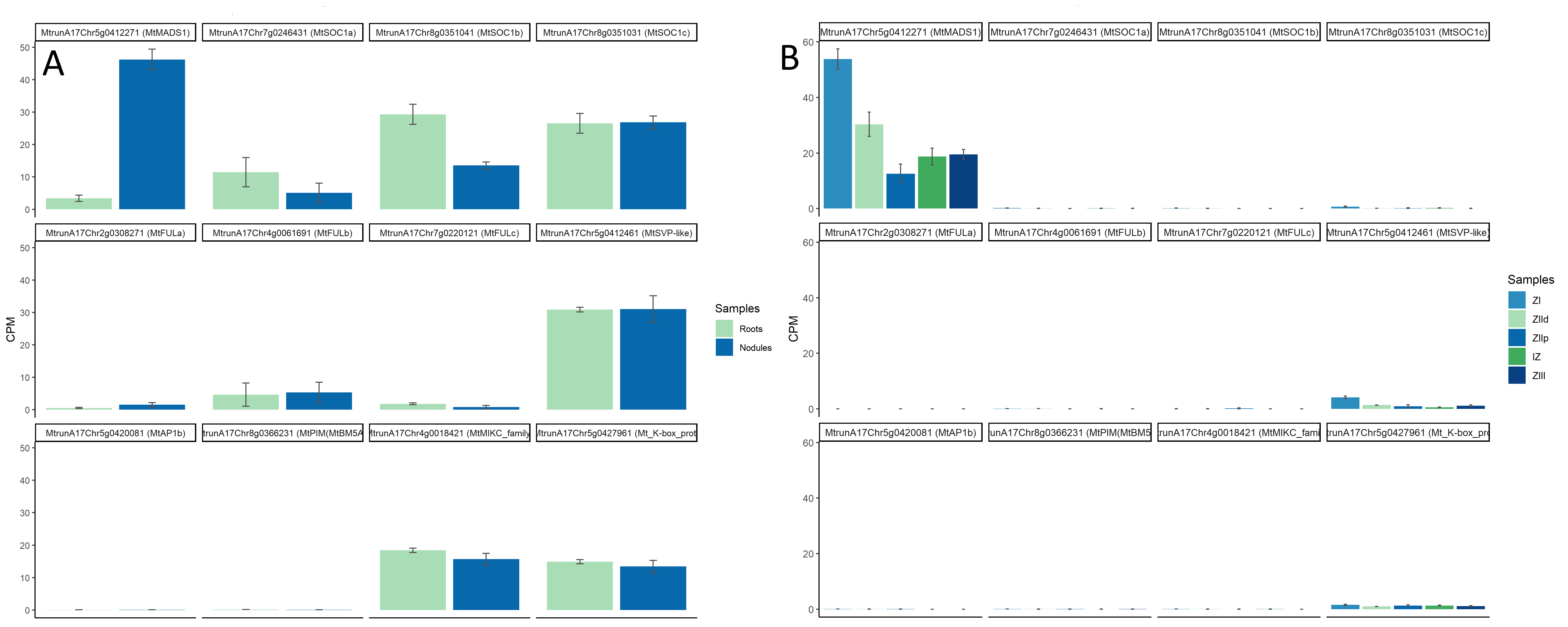

Supplement: Supplementary file 1 [file plants-12-00657-s001.zip › Figure S3.tiff]

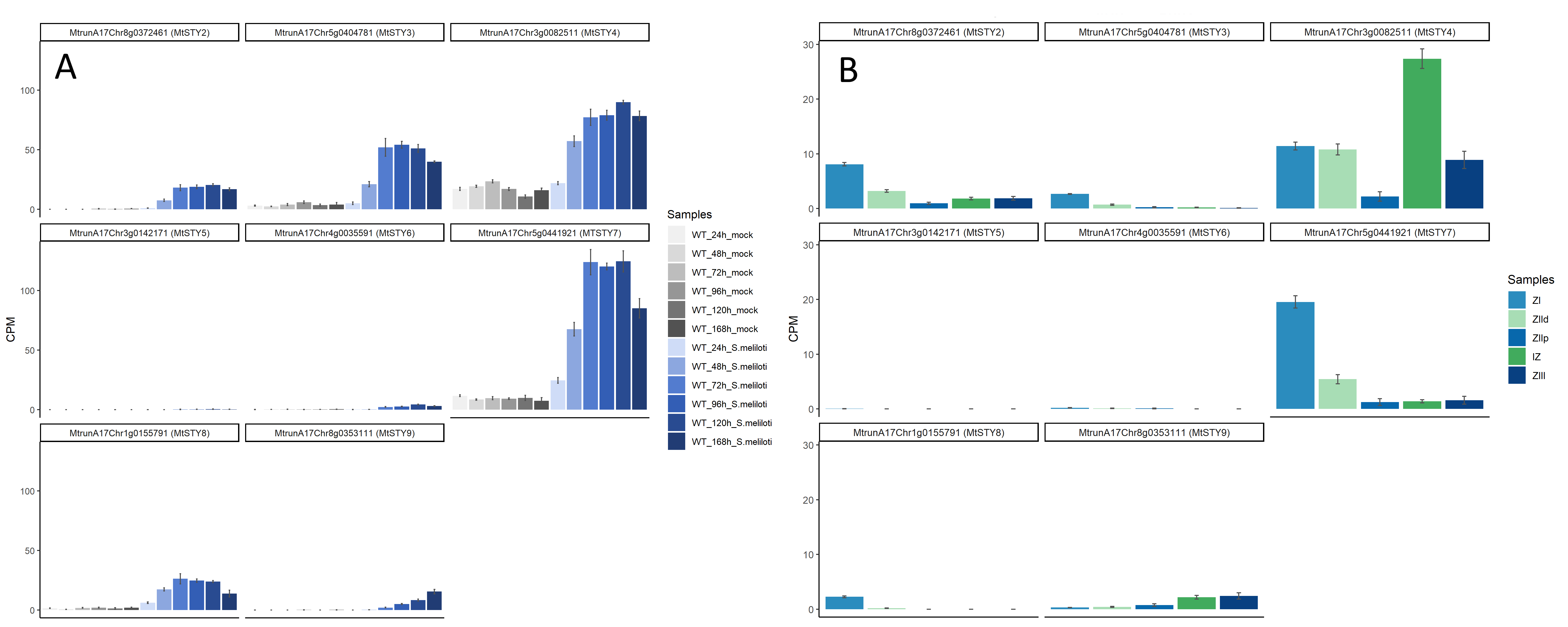

Supplement: Supplementary file 1 [file plants-12-00657-s001.zip › Figure S4.tiff]

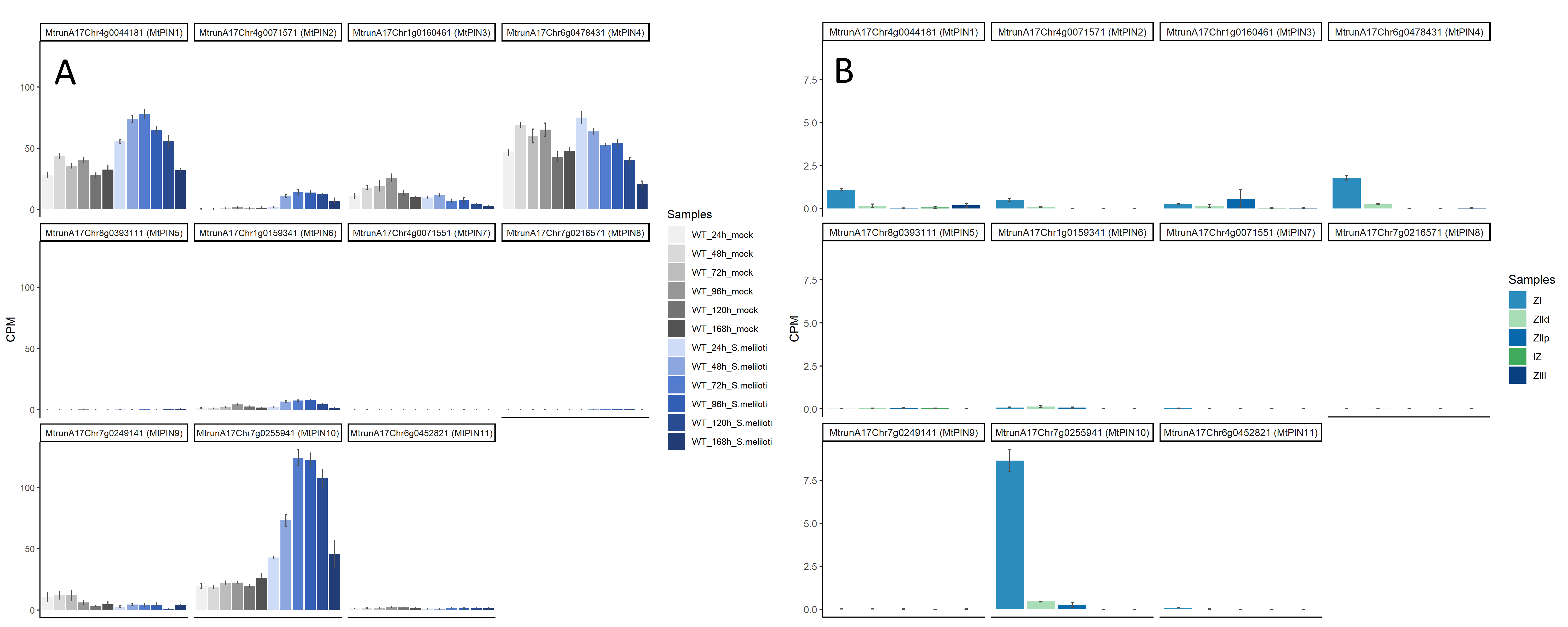

Supplement: Supplementary file 1 [file plants-12-00657-s001.zip › Figure S5.tiff]
